# Development of *Bt* Rice and *Bt* Maize in China and Their Efficacy in Target Pest Control

**DOI:** 10.3390/ijms17101561

**Published:** 2016-10-18

**Authors:** Qingsong Liu, Eric Hallerman, Yufa Peng, Yunhe Li

**Affiliations:** 1 State Key Laboratory for Biology of Plant Diseases and Insect Pests, Institute of Plant Protection, Chinese Academy of Agricultural Sciences, Beijing 100193, China; qingsongliu00@gmail.com (Q.L.); yfpeng@ippcaas.cn (Y.P.); 2 Department of Fish and Wildlife Conservation, Virginia Polytechnic Institute and State University, Blacksburg, VA 24061-0321, USA; ehallerm@vt.edu

**Keywords:** *Bacillus thuringiensis*, Cry proteins, target insects, commercialization, ELISA

## Abstract

Rice and maize are important cereal crops that serve as staple foods, feed, and industrial material in China. Multiple factors constrain the production of both crops, among which insect pests are an important one. Lepidopteran pests cause enormous yield losses for the crops annually. In order to control these pests, China plays an active role in development and application of genetic engineering (GE) to crops, and dozens of GE rice and GE maize lines expressing insecticidal proteins from the soil bacterium *Bacillus thuringiensis* (*Bt*) have been developed. Many lines have entered environmental release, field testing, and preproduction testing, and laboratory and field experiments have shown that most of the *Bt* rice and *Bt* maize lines developed in China exhibited effective control of major target lepidopteran pests on rice (*Chilo suppressalis*, *Scirpophaga incertulas*, and *Cnaphalocrocis* *medinalis*) and maize (*Ostrinia furnacalis*), demonstrating bright prospects for application. However, none of these *Bt* lines has yet been commercially planted through this writing in 2016. Challenges and perspectives for development and application of *Bt* rice and maize in China are discussed. This article provides a general context for colleagues to learn about research and development of *Bt* crops in China, and may shed light on future work in this field.

## 1. Introduction

Rice (*Oryza sativa* L.) and maize (corn) (*Zea mays* L.) are important cereal crops in China. Rice serves as a staple food for more than half of the world’s people [1], and maize serves as food, feed, and industrial material [2,3]. The yields of rice and maize have increased significantly since the adoption of high-yielding selectively bred and hybrid varieties [4], and have reached nearly 208 and 215 million tons in 2014, respectively, in China [5]. However, with the growth of China’s population and the steady decrease in the amount of arable land, the yield of the two crops must increase to meet the increasing demand [1,6]. Multiple factors constrain the production of rice and maize, among which insect pests are an important one. The major insect pests on the two crops are lepidopterans. On rice, four major lepidopteran pests-rice striped stem borer *Chilo suppressalis* (Family Crambidae), yellow stem borer *Scirpophaga incertulas* (Family Crambidae), pink stem borer *Sesamia inferens* (Family Noctuidae) and rice leaf roller *Cnaphalocrocis medinalis* (Family Crambidae)—cause severe yield losses annually [7]. It has been estimated that the rice stem borers cause an annual 3.1% loss of yield nationally, equivalent to an economic loss of $US 1.9 billion each year in China [8]. The major lepidopteran pests on maize are *Ostrinia furnacalis* (Family Crambidae), *Mythimna separata* (Family Noctuidae), and *Helicoverpa armigera* (Family Noctuidae), causing 10% of yield loss in spring maize, 20%–30% in summer maize, and over 30% with heavy infestations, resulting in a huge economic loss every year [3,9]. Aside from the direct yield losses, maize infestation by lepidopteran pests may result in the production of fumonisins, mycotoxins that lower the quality of maize and may pose negative effects on livestock [3]. Multiple strategies have been developed to control rice and maize pests, with chemical insecticide application as the main measure [9,10]. However, the application of chemical insecticides has brought a series of problems, such as air, water, and soil pollution, food contamination, the resurgence of resistant herbivores, and reduction of populations of natural enemies of the crop pests.

Genetic engineering (GE) technology provides a powerful and clean tool for insect pest control. Since the first commercialization in the United States in 1996, GE crops have been widely and rapidly adopted worldwide [11]. Among the GE crops in commercial production, those expressing insecticidal proteins from the soil bacterium *Bacillus thuringiensis* (*Bt*), generally called *Bt* crops, are an important subset. To improve agricultural productivity, China has played an active role in the development and application of GE crops since the 1980s [3,12,13]. A huge research project, called the National GMO New Variety Breeding Program, was initiated in China in 2008, which is expected to invest $US 3.5 billion through 2020 [12,13]. With the massive financial support, great progress has been made in research and development of GE crops, and a large number of GE crop events and varieties have been obtained in China, with traits including herbicide tolerance, insect resistance, drought resistance, stress tolerance, heightened quality and high yield [1,3,14]. In particular, dozens of *Bt* rice and *Bt* maize lines have been developed, and many have entered environmental release field testing, and preproduction testing [13]. Critically, two *Bt* rice lines, Huahui 1 and *Bt* Shanyou 63, have obtained biosafety certificates for commercial production, although they have not been grown commercially to date.

In the current article, we summarize the development of *Bt* rice and *Bt* maize and analyze the expression levels of Cry protein and their efficacy in target pest control. Meanwhile, the prospects of commercialization of *Bt* rice and *Bt* maize in China are discussed, with the objective of providing a general sense of research and development of *Bt* crops in China.

## 2. *Bt* Rice

### 2.1. Bt Rice Lines Developed

The first insect-resistant genetically engineered (IRGE) rice line expressing a *Bt* delta-endotoxin gene driven by the *CaMV 35S* promoter was developed in 1989 [15], and so far dozens of *Bt* rice lines have been produced in China. Most of the *Bt* rice lines were developed by public-sector scientists from Huazhong Agricultural University, Fujian Academy of Agricultural Sciences, Chinese Academy of Sciences, Chinese Academy of Agricultural Sciences, and Zhejiang University. These *Bt* rice lines can be divided into three categories, namely: (i) lines containing a single *Bt* gene, such as *cry1Ab* in the Kemingdao (KMD) and mfb-MH86 lines; *cry1Ac* in AC-1, E10, and E54; *cry1C* in T1C-19, and C-54; *cry2A* in T2A-1, T2A-2, T2A-3, and T2A-4; and *cry9C* in 9C-1, 9C-2, 9C-3, 9C-4, and 9C-5; (ii) containing a fusion *Bt* gene, such as the *cry1Ab/1Ac* fusion gene in TT51-1 (Huahui 1), TT9-3, and *Bt* Shanyou 63; and the *cry1Ab*/*vip3H* gene in G6H-1, G6H-2, G6H-3, G6H-4, G6H-5, and G6H-6; and iii) containing stacked insecticidal genes such as *cry1Ac* and modified *CpTI* (cowpea trypsin inhibitor) in MSA, MSB, and Kefeng6 (Table 1). In addition, some *Bt* rice lines were stacked with other types of transgenes, such as *bar* for herbicide tolerance, and *Xa21* for disease resistance (Table 1). In the development of *Bt* rice lines, China made great efforts for independent innovation, and also took an active part in international cooperation. For example, KMD was developed by Zhejiang University in collaboration with the University of Ottawa, and Huahui 1 and *Bt* Shanyou 63 were developed by Huazhong Agricultural University in collaboration with the International Rice Research Institute [16]. *Agrobacterium*- and gene gun-mediated transformations are commonly used for *Bt* rice development, and the promoters used for driving the expression of *Bt* genes include *ubiquitin*, rice *rbcS* (small subunit of ribulose-1, 5-bisphosphate carboxylase/oxygenase) promoter, and *Actin1* (Table 1).

### 2.2. Bt Protein Expression in Rice Plants

Efficient stable expression of *Bt* proteins in plants is the basis for high efficacy in controlling target pests, and also is important for delaying the development of *Bt* resistance of target pests [22,26,64]. At an early stage of *Bt* crop development, unmodified *Bt* genes were directly introduced into plants, resulting in low levels of protein expression, which were not sufficient for efficiently controlling target pests [65]. Subsequently, *cry1A*(*b*) and *cry1A*(*c*) genes were modified before being introduced into cotton [66], tobacco, and tomato [67], leading to increased expression of Cry proteins and thus improved resistance to target insect pests, and led to realization of commercial use of *Bt* crops.

To detect the expression of *Bt* proteins in GE plants, multiple immunological methods were developed, including Bradford's method, Western blotting, immunohistochemical staining, lateral flow strips, and enzyme-linked immunosorbent assay (ELISA) [26]. Among these methods, ELISA is a relatively efficient detection method, offering simple, fast, and reliable protein determination, and it has been widely used for qualitative and quantitative analyses of Cry proteins in *Bt* plants [22,26].

Using the ELISA method, various studies have been conducted to measure the levels of Cry proteins in *Bt* rice plants (Table 1). In general, the levels of Cry protein in different plant tissues varied significantly, with the highest level in leaves, followed by stems, roots and seeds. An exception was found in *Bt* rice lines (G6H-1, G6H-2, G6H-3, G6H-4, G6H-5, and G6H-6), in which the content of Cry1Ab was higher in stems than in leaves. Throughout the growing season, different *Bt* rice lines display different expression dynamics of Cry proteins. To sum up, expression dynamics can be classified into three patterns: (i) declining expression, with high expression at an early stage (seedling stage) with gradually decreased levels at later stages; this is the most frequent pattern not only in *Bt* rice, but also in *Bt* cotton, soybean and maize [22,25,26,34,38,56,62,68]; (ii) increasing expression, namely low expression at an early stage with increased expression levels at later stages [17,69]; and (iii) relatively consistent expression [26,34,70]. Interestingly, relatively high Cry protein expression levels were detected in lines expressing *cry2Aa* or *cry9C* genes among the *Bt* rice lines. The actual mechanism for this phenomenon is unclear, although it was inferred that it might be associated with the high contents of the bases G and C in these genes, since it was found that under the same conditions (the identical promoter, terminator, binary expression vector, recipient variety, and similar selection criteria), genes containing higher G and C content could be more highly expressed in plants [31].

In *Bt* rice, constitutive promoters such as *ubiquitin*, and *Actin1* are widely used to express *Bt* genes, resulting in *Bt* proteins being produced in the whole plant (Table 1). However, in order to reduce the potential risk of *Bt* resistance developed by target insect pests and due to consumer safety concerns, tissue-specific promoters have started to be used for *Bt* rice development. For example, the green tissue-specific promoters rice *rbcS* and *pGreen* have been used in the rice lines RJ-5 and S21, respectively, resulting in the content of Cry proteins being only 0.0026 μg/g in endosperm [38] and 0–0.0076 μg/g [51] in seeds. It seems that such tissue-specific promotors have prospects for wider application.

### 2.3. Target Pest Control

Laboratory and field experiments have been extensively conducted to test the efficacy of the *Bt* rice lines against target pests in China. Under laboratory conditions, bioassays were conducted in which the stems or leaves were cut from rice plants and fed to different instars of target pests, and the mortalities of the tested pests were taken as an indicator of the resistance of *Bt* rice plants to target pests. However, under field conditions, the percentage of plants with rolled leaves (mainly for *C. medinalis*), dead-heart (mainly for *C. suppressalis*), and white-head (mainly for *S. incertulas*) were taken as indicators of the species-specific damage characteristics caused by different caterpillars.

Multiple lepidopteran pests were tested for their susceptibility to *Bt* rice, with the focus on *C. suppressalis*, *S. incertulas*, and *C. medinalis* due to their severity in rice fields (Table 1). Laboratory bioassays indicated that *Bt* rice lines showed high resistance to young (<2nd-instar) caterpillars, and the efficacy of resistance decreased significantly with increasing age of the insects. For example, the corrected mortalities of 1st- to 6th-instar *C. suppressalis* in 7-day bioassays feeding on rice expressing *cry1Ac* + *CpTI* genes were 89.6%, 87.1%, 72.37%, 50.0%, 26.6%, and 0%, respectively [59]. Since the expression of *Bt* proteins in rice normally shows a declining tendency with the growth of rice plants, the resistance to target pests generally declined over the growth stages of rice plants; at mature stage most *Bt* rice lines showed relatively poor anti-pest efficacy [34,55,56,71]. However, *Bt* rice lines such as *Bt*-DL, mfb-MH86, and Huahui 1 showed high and consistent pest resistance throughout the growing season due to the stable expression of *Bt* protein in rice plants [22,26,34].

Field trials also showed that many *Bt* rice lines exhibited high resistance to target pests, providing 90%–100% control of stem borers and 80%–100% control of leaf-folders (Table 1). Similar to laboratory results, most *Bt* rice lines performed better in target insect pest control during the early growing season, and poorer control later in the growing season, with the exception of *Bt* rice lines *Bt*-DL, mfb-MH86, and Huahui 1, which exhibited excellent control of target pests throughout the rice-growing season.

As mentioned above, the efficacy of *Bt* rice lines for controlling target pests is positively correlated with the level of expression of Cry proteins in plant tissues [22,26,56,62]. However, high Cry protein expression does not always exert high insect resistance, since resistance is also related to the types of *Bt* proteins produced in rice plants; different *Bt* proteins showed significantly different toxicity to different target species. For example, although some rice lines contained Cry9C or Cry2A proteins at much higher levels than Cry1C and Cry1A proteins in some other rice lines, these lines showed equivalent or even lower resistance to target pests than the *cry1C* or *cry1A* rice lines [31]. This phenomenon can be explained by the results of Jiao et al. (2016) [72], showing susceptibility of *C. suppressalis* larvae to five Cry proteins in the order Cry1Ca > Cry1Ab > Cry1Ac > Cry2Aa > Cry1Fa. By comparison, Cry1Ab, Cry1Ac and Cry1C proteins seem to be ideal insecticidal proteins for incorporation into rice to control lepidopteran rice pests. Further, these three *Bt* proteins have relatively low toxicity to silkworm *Bombyx mori* (Lepidoptera: Bombycidae) larvae, which we are trying to protect [72].

Various studies have shown that *Bt* rice could provide effective control of major lepidopteran pests. However, both laboratory and field studies have pointed out that these rice lines show relatively low resistance to *S. inferens* especially at late growth stages of rice [46,50,55,62]. Laboratory study also confirmed that *S. inferens* exhibited significantly lower susceptibility to Cry1A proteins than *C. suppressalis*, which suggests that *S. inferens* is likely to develop resistance to *Bt* rice after commercial planting [73]. Therefore, more attention should be paid to those species in development of insect resistance management strategies for *Bt* rice.

## 3. *Bt* Maize

### 3.1. Bt Maize Lines Developed

Development of *Bt* maize started in the late 1980s in China, but moved relatively slowly during the initial stage. Greater progress was achieved in the past decade, especially after the initiation of the National GMO New Variety Breeding Program in 2008. To date, over a dozen *Bt* maize lines have been obtained (Table 2). Most were developed by public-sector scientists from the Chinese Academy of Agricultural Sciences, Zhejiang University, China Agricultural University, and Shandong University. In recent years, several agricultural biotechnology companies became involved in *Bt* maize development, for example the DBN Sci-tech Group, China National Seed Group Co., Ltd., (Beijing, China) and Beijing Origin Seed Technology, Inc. (Beijing, China) Similar to *Bt* rice, all *Bt* maize lines developed in China express *cry1* and/or *cry2* genes targeting lepidopteran pests. Most of the *Bt* maize lines contain a single *Bt* gene, such as *cry1Ac* in the BT-799 and Zhengdan958K lines, *cry1Ie* in IE09S034, and *cry1Ah* in G186 (Table 2). Some *Bt* maize lines contain a fusion *Bt* gene, such as *cry1Ab*/*cry2Aj* in Shuangkang 12-5, and *cry1Ah/cry1Ie* in HIF21 (Table 2). In addition, there were several *Bt* maize lines stacked with the *epsps*, *bar*, or *G10evo-epsps* genes, thereby exhibiting both pest resistance and herbicide tolerance (Table 2). *Agrobacterium*-, gene gun- and pollen tube-mediated techniques were commonly used for *Bt* maize transformation. The promoters used in *Bt* maize mainly include *pZmUbi-1* (*Zea mays* polyubiquitin-1), *P35S*, and *CaMV 35S* (Table 2).

### 3.2. Bt Protein Expression in Maize Tissues

The same as for *Bt* rice, the highest *Bt* protein level in maize normally was detected in leaves, followed by stems and roots, and the least in seeds [78,83,90,91,92]. There also was high content of Cry protein detected in husks, kernels, tassels, and silks of *Bt* maize [75,86,87]. In general, it was reported that Cry protein concentrations in *Bt* maize tissues decreased with maize growth [90,91]. However, there also were cases when Cry protein content in maize tissues increased with age. For example, the contents of mCry1Ac protein in the leaves, stems and roots of BT-799 increased with the growth of the plants, and reached a peak at the seed maturation stage [92]. The expression dynamics of Cry protein were investigated in several *Bt* maize lines. For example, the content of Cry1Ah in the leaves of line G186 increased through early stages, reaching the peak at the heading stage, but then decreased at later stages [83]. The content of CryFLIa (modified Cry1Ab) protein in leaves of HiII showed an increasing trend before the 8-leaf stage, and decreased at the heading stage, but increased again at the filling stage [74]. The expression of *Bt* proteins in plants is a complicated process which can be affected by the genetic background of different recipient cultivars, promoters, transformed genes, transformation methods, growth environment, physiological conditions, and the plant’s energy resources.

### 3.3. Target Pest Control

Studies were conducted to test pest-control efficacy of *Bt* maize, mainly focusing on the target lepidopteran pest *O. furnacalis*. In laboratory studies, the mortality of *O. furnacalis* larvae was evaluated when fed leaves, tassels, husks, silks, spikes, and kernels from *Bt* maize as compared to non-*Bt* control maize. The results showed that the majority of the currently developed *Bt* maize lines caused over 85% mortality of *O. furnacalis* larvae; a few *Bt* maize lines caused 100% mortality when *O. furnacalis* neonates were fed *Bt* maize leaves (Table 2). It seems that *Bt* maize has poorer control of *H. armigera* than *O. furnacalis.* For example, the *Bt* maize line IE09S034 caused over 85% mortality of *O. furnacalis* larvae, but only 50% mortality of *H. armigera* (Table 2). In field trials, *Bt* and non-*Bt* control maize plants were artificially infested with *O. furnacalis* neonates, and a few days later, leaf damage ratings (LDR) were assessed (Table 2). According to the criteria described by He et al. (2000) [89] (for details see the footnotes under Table 2), the LDRs by *O. furnacalis* on all test *Bt* maize lines were below 3, and the resistance levels were characterized as “highly resistant” or “resistant”. Several *Bt* maize lines, such as BT-X, Shuangkang 12-5, V3, HGK60, G186, and N30, exhibited excellent control of *O. furnacalis*, with LDRs less than 1.5. In general, the *Bt* maize lines expressing Cry1Ab or Cry1Ac protein performed better in controlling *O. furnacalis* larvae, suggesting that both *cry1Ab* and *cry1Ac* genes are ideal for maize transformation for controlling lepidopteran pests.

## 4. Conclusions and Future Perspectives

Both laboratory and field studies showed that multiple *Bt* rice and *Bt* maize lines developed in China expressed effective control of target lepidopteran pests (Table 1 and Table 2). In addition, many studies have been conducted to assess the ecological and food safety of *Bt* rice and *Bt* maize. While we do not address these issues in the current review, the results indicate that the currently developed *Bt* rice and *Bt* maize pose negligible risk to the environment and human health [1,93]. Thus, we conclude that compared to conventional pesticide-treated crop production, planting of *Bt* rice and *Bt* maize should be safer to the consumer and more environmentally friendly. However, as mentioned above, some lepidopteran pests, such as *S. inferens* on rice and *H. armigera* on maize cannot be efficiently controlled by the current *Bt* rice and *Bt* maize lines, and scientific insect resistance management strategies should be developed prior to commercial cultivation of these *Bt* plants [64].

The currently developed *Bt* rice and *Bt* maize lines are all for controlling lepidopteran pests. However, other insects, such as planthoppers on rice and aphids and spider mites on maize, also cause considerable economic losses annually. Unfortunately, there are as yet no optimal genes for use to control such piercing and sucking insects. Investigation of such genes is an urgent issue. Once such genes are identified, they should be stacked with Lepidopteran-resistance genes for rice and maize transformation.

Although excellent *Bt* rice and *Bt* maize lines have been obtained, no *Bt* crops have yet been commercially planted in China. An important milestone for *Bt* rice came in 2009 when the Ministry of Agriculture of China issued biosafety certificates for commercial production of the *Bt* rice lines Huahui 1 and *Bt* Shanyou 63 in Hubei province, and in 2014 when the biosafety certificates were renewed. The delay in commercial use of *Bt* rice is largely caused by low public acceptance due to extreme concerns about the food safety of GE crops and the low scientific literacy of the public about GE crops more generally [1]. This situation is not particular to China, and occurs in many countries, for example in European Union countries, in which GE crop products are even less accepted. To change this condition, more work should be done by government and non-governmental organizations, such as developing targeted and well-funded educational programs and increasing public dialog on GE crops [1,94,95]. Scientists, in particular mainstream scientists working with genetically modified organisms (GMOs), should be actively engaged in risk communication regarding GMOs [1,94,95]. Public dialog and risk communication of GMOs can be accomplished through television, the Internet using media such as Weibo (the Chinese version of Twitter) and WeChat (the most universal communication applications recently in China), newspapers, and periodicals [95,96]. In addition, it is important for agricultural oversight agencies to enhance their ability to supervise and regulate GMO biosafety, since any potential incidents associated with GMO biosafety may impair public confidence in the biosafety on GMOs [1].

## Figures and Tables

**Table 1:** Insect-resistant *Bt* rice lines and their efficacy on target lepidopteran pests in China.

Insecticidal Proteins	Plant Lines	Promoter; Method of Transformation	Parental Line or Cultivar	Expression Level of *Bt* Protein ^a^	% Efficacy on Target Pests		References
					In Laboratory	In Field	
Cry1Ab	KMD1	*Ubiquitin*; *Agrobacterium*-mediated	Xiushui 11 (*japonica*)	3.74–7.50 μg/g in stems FW; 3.78–9.13 μg/g in leaves FW; 12.78 μg/g in pollen DW	100% for 1st- or 3rd-instar larvae of 8 lepidopteran species *; 78% (4th-instar), and 68% (5th-instar) for *C. medinalis*	100% for *C. suppressalis*, *S. incertulas* and *C. medinalis*	[17,18,19,20,21]
	KMD2	*Ubiquitin*; *Agrobacterium*-mediated	Xiushui 11 (*japonica*)	4.32–8.84 μg/g in stems FW; 3.97–8.29 μg/g in leaves FW; 31.37 μg/g in pollen DW	100% for *C. suppressalis*	100% for *C. suppressalis*	[17,18,21]
	mfb-MH86	*Ubiquitin*; *Agrobacterium*-mediated	Minghui 86 (*indica*)	9.71–34.09 μg/g in leaves DW; 7.66–18.51 μg/g in stems DW; 1.95–13.40 μg/g in roots DW	100% for *C. suppressalis*	-	[22]
	T_1Ab_-10	*Ubiquitin*; *Agrobacterium*-mediated	Minghui 63 (*indica*)	7.54 μg/g in leaves FW	-	100% for *C. medinalis*, 98.2%–100% for *C. suppressalis*, 98.9%–100% for *S. incertulas*	[23]
	-	Rice *rbcS* promoter; *Agrobacterium*-mediated	Zhejing22 (*japonica*)	1.66–3.31 μg/g in leaves FW; 0.11–0.17 μg/g in seeds FW	-	-	[24]
	DL (hybrid)	-	-	2.49–16.13 μg/g in leaves, stems and roots FW	91.7%–100% for *C. suppressalis*	97.5%–100% for *C. suppressalis*	[25,26]
	-	*Actin1*; Gene gun-mediated	Zhongguo 91 (*japonica*)	-	100% for *C. suppressalis*	-	[27]
	-	*Ubiquitin*; *Agrobacterium*-mediated	Zhongguo 91 (*japonica*)	-	-	>99% for *C. suppressalis*	[28,29]
Cry1Ac	-	*Ubiquitin*; *Agrobacterium*-mediated	Xiushui 11 (*japonica*)	-	100% for *C. suppressalis*, *S. incertulas*, *C. medinalis*, and *Psara licarisalis*	-	[30]
	Ac-1, Ac-2	*Ubiquitin*; *Agrobacterium*-mediated	Minghui 63 (*indica*)	11.09 (Ac-1), and 14.48 (Ac-2) μg/g in leaves FW	100% for *S. incertulas*	100% for *C. suppressalis*	[31]
	P6, H7	*Ubiquitin*; *Agrobacterium*-mediated	Guanglingxiangjing (*japonica*)	0.025%–0.10% in leaves	100% for 2nd-instar *C. suppressalis* and *C. medinalis*	100% for *C. medinalis*	[32]
	E10, E19	*Ubiquitin*; *Agrobacterium*-mediated	Wuxiangjing9 (*japonica*)	0.025%–0.10% in leaves	100% for 2nd-instar *C. suppressalis* and *C. medinalis*	100% for *C. medinalis*	[32]
Cry1C	T1C-19	*Ubiquitin*; *Agrobacterium*-mediated	Minghui 63 (*indica*)	Up to 3.65 μg/g in leaves DW	85%–100% for *C. suppressalis*	94.8%–100% for *C. medinalis*; 99.98%–100% for *C. suppressalis*	[33,34,35,36,37]
	RJ-5	Rice *rbcS* promoter; *Agrobacterium*-mediated	Zhonghua 11 (*japonica*)	0.87 μg/g in leaves FW; 0.0026 μg/g in endosperm FW	-	97.9% for stem borers, and 99.4% for leaf folders	[38]
	-	*Ubiquitin*; *Agrobacterium*-mediated	Hanhui 3 (*indica*)	0.46–2.11 μg/g in leaves FW	-	100% for *C. medinalis*	[39]
	C-6	Rice *rbcS* promoter; *Agrobacterium*-mediated	Jijing 88 (*japonica*)	2.42 μg/g in leaves FW	-	97.1% for *C. suppressalis*	[40]
	C-54	Rice *rbcS* promoter; *Agrobacterium*-mediated	Jili 518 (*japonica*)	2.27 μg/g in leaves FW	-	95.9% for *C. suppressalis*	[40]
Cry2A	T2A-1, T2A-2, T2A-3, T2A-4	*Ubiquitin*; *Agrobacterium*-mediated	Minghui 63 (*indica*)	9.65–12.11 μg/g in leaves FW	100% for *S. incertulas*	92.5%–94.6% for *S. incertulas*; 95.8%–99.0% for *C. medinalis*	[41]
	T2A-1	*Ubiquitin*; *Agrobacterium*-mediated	Minghui 63 (*indica*)	Up to 87.25 μg/g in leaves DW; 33.5 μg/g in pollen DW	55.6%–100% for *C. suppressalis*; 64.69% (1st-instar), and 64.92% (3rd-instar) for *C. medinalis*	95.7%–100% for *C. medinalis*; 99.9%–100% for *C. suppressalis*	[34,35,37,42]
	2A-1, 2A-2, 2A-3	*Ubiquitin*; *Agrobacterium*-mediated	Minghui 63 (*indica*)	109.35–138.75 μg/g in leaves FW	100% for *S. incertulas*	84.6%–91.7% for *C. suppressalis*	[31]
	B2A68	*Ubiquitin*; *Agrobacterium*-mediated	D68 (*indica*)	10.45–26.84 μg/g in leaves FW	100% for *C. suppressalis*	-	[43]
Cry9C	9C-1, 9C-2, 9C-3, 9C-4, 9C-5	*Ubiquitin*; *Agrobacterium*-mediated	Minghui 63 (*indica*)	655.46, 324.55, 166.83, 365.07, and 182.61 μg/g in leaves FW, respectively	100% for *S. incertulas*	100%, 100%, 91.3%, 96.2%, and 91.7% for *C. suppressalis*, respectively	[31]
Cry1Ab/1Ac	TT51-1 (Huahui 1)	*Actin1*; Gene gun-mediated	Minghui 63 (*indica*)	20 μg/g soluble protein in leaves; 1.39 μg/g in leaves FW; 0.78 μg/g in stems FW; 0.87 μg/g in roots FW; Up to 8.07 μg/g in roots DW	91.7%–100% for *C. suppressalis*; 100% for *S. incertulas*	84.8%–100% for *C. medinalis*; 91.4%–95.7% for *S. incertulas*	[34,44,45]
	TT9-3, TT9-4	*Actin1*; Gene gun-mediated	IR72 (*indica*)	Up to 0.01% in leaves	-	>90% for *S. inferens*, *C. suppressalis*, *S. incertulas*, *C. medinalis*, and *N. Anescens*	[46,47]
	Shanyou 63 (hybrid)	-	-	Up to 7.55 μg/g in leaves FW; 1.11 μg/g in stems FW; 0.84 μg/g in roots FW	67.9% for *C. suppressalis* (3rd-instar); 100% (1st- and 3rd-instar), and 85% (5th-instar) for *S. inferens*	92.5%–100% for *C. suppressalis*; 88%–100% for *C. medinalis*, 98.9%–99.62% for *S. incertulas*	[26,37,44,45,48,49]
Cry1Ab/Vip3H	G6H1, G6H2, G6H3, G6H4, G6H5, G6H6	*Ubiquitin*; *Agrobacterium*-mediated	Xiushui 110 (*japonica*)	Cry1Ab: 0.001-0.038% in leaves, 0.006-0.073% in main stems	100% for *C. suppressalis* and *S. inferens*	100% of G6H1, G6H2, and G6H6 for *C. suppressalis* and *S. inferens*	[50]
Cry1Ac/Cry1I-like	S21	*pGreen*; *Agrobacterium*-mediated	Xiushui 134 (*japonica*)	1.05–1.51 μg/g in leaves FW; 0.67–1.16 μg/g in stems FW; 0–0.0076 μg/g in seeds FW	-	100% for *C. suppressalis* and *C. medinalis*	[51]
Cry1Ac + CpTI	MSA	*Actin1*; Gene gun-mediated	Minghui 86 (*indica*)	Up to 1.2 μg/g in leaves FW; up to 0.28 μg/g in stems FW; 0.013 μg/g in seeds FW	92.8%–100% before the filling stage, 60% after the filling stage for *C. suppressalis*; 98.1%–100% for *C. medinalis*; 34.0%–72.8% for *S. inferens*	80%–100% for *C. medinalis*; 99.9% for *C. suppressalis;* 93.3% for *S. inferens*	[52,53,54,55,56]
	MSB	*Actin1*; Gene gun-mediated	Minghui 86 (*indica*)	Up to 0.96 μg/g in leaves FW; up to 0.34 μg/g in stems FW; 0.017 μg/g in seeds FW	79.3%–100% before filling stage, 60% after the filling stage, and 64% at maturing stage for *C. suppressalis*; 94.3%–100% for *C. medinalis*; 41.3%–62.5% for *S. inferens*	98.5%–100% for *C. suppressalis*; 80%–100% for *C. medinalis*; 93.3%–95.3% for *S. inferens*	[52,53,54,55,56,57,58]
	Minghui 86^CpTI+*Bt*^	*Actin1*; Gene gun-mediated	Minghui 86 (*indica*)	-	89.6%, 87.1%, 72.37%, 50.0%, 26.6%, 0% for 1st- to 6th- instar *C. suppressalis*	99.03%–100% for *S. inferens*, 97.4%–100% for *C. suppressalis*, 98.63%–100% for *S. incertulas*, 92.5%–99.17% for *C. medinalis*	[59,60]
	Kefeng6 (KF6)	*Actin1*; Gene gun-mediated	Minghui 86 (*indica*)	Up to 7.55 μg/g in leaves FW	54.2%–100% for *C. suppressalis*, 0%–100% for *S. inferens*, 100% for *C. medinalis*	90%–99.4% for *C. suppressalis*, 83.3%–100% for *S. inferens*, 93.3%–100% for *C. medinalis*	[26,61,62,63]
	IIYouKF6 (hybrid)	-	KF6, IIYouMH86	Up to 2.16 μg/g in leaves FW; up to 1.65 μg/g in main stems FW	11.36%–100% for *S. inferens*; 90.2%–100% for *C. medinalis*	93.3%–100% for *C. medinalis*	[61,62,63]

^a^ % of total soluble protein or μg/g tissue fresh weight (FW) or dry weight (DW); “-” denotes “unclear”; * *Chilo suppressalis*, *Scirpophaga incertulas*, *Cnaphalocrocis medinalis*, *Herpitogramma licarisalis*, *Sesamia inferens*, *Naranga anescens*, *Mycalesis gotama*, and *Parnara guttata*.

**Table 2:** Insect-resistant *Bt* maize lines and their efficacy on target lepidopteran pests in China.

Insecticidal Proteins	Plant Lines	Promoter; Method of Transformation	Recipient Cultivar	Expression Level of *Bt* Protein ^a^	Efficacy on Target Lepidopteran Pests		References
					In Laboratory	In Field ^b^	
Modified Cry1Ab	-	*pZmUbi-1*; *Agrobacterium*-mediated	HiII	0.30–0.47 μg/g in leaves FW	78% of leaves for *O. furnacalis* in 5-day bioassays	0.14 survivors, 2.43 tunnels/plant, 3.64 cm tunnel length/plant	[74]
mCry1Ac	BT-799	*CaMV 35S*; Gene gun-mediated	Zheng 58	0.77 μg/g in leaves FW; 0.23 μg/g in silks DW; 0.30 μg/g in husks DW; 0.15 μg/g in young kernels DW; 0.059 μg/g in pollen DW	-	Leaf damage ratings (LDR) below 2 for *O. furnacalis*	[75,76,77]
	Zhengdan958K	-	Zhengdan 958	-	100% of whorl leaves, 83.3% of silk, 97.2% of husk, and 63.5% of young kernel for *O. furnacalis*	-	[75]
Cry1Ac	BT-X	*CaMV 35S*; -	HiII × H99	0.087–0.23 μg/g in whorl leaves FW; 0.044 μg/g in silks FW	84.7%–97.2% of whorl leaves for *O. furnacalis*	LDR was 1.15 for *O. furnacalis*	[78]
	BT-38	*CaMV 35S*; -	Zheng 58	0.44 μg/g in whorl leaves FW	98.6% of whorl leaves for *O. furnacalis*	-	[78]
	BT-181	*CaMV 35S*; -	Zheng 58	0.42 μg/g in whorl leaves FW	97.2% of whorl leaves for *O. furnacalis*	-	[78]
	BT-105	*CaMV 35S*; -	Chang 7-2	0.42 μg/g in whorl leaves FW	100% of whorl leaves for *O. furnacalis*	-	[78]
Cry1AcM	C1, C2, C3	*pZmUbi-1*; *Agrobacterium*-mediated	Chang 7-2	-	LDR was below 2.08, >80% of kernels, and >90% of husks for *O. furnacalis*	LDR was below 1.91, >80% of kernels, and >90% of husks for *O. furnacalis*	[79]
	Z1, Z2, Z3	*pZmUbi-1*; *Agrobacterium*-mediated	Zheng 58	-	LDR was below 2.07, >80% of kernels, and >90% of husks for *O. furnacalis*	LDR was below 1.50, >80% of kernels, and >90% of husks for *O. furnacalis*	[79]
	Q1, Q2, Q3	*pZmUbi-1*; *Agrobacterium*-mediated	Qi 319	-	LDR was below 2.0, >80% of kernels for *O. furnacalis*	LDR was below 1.11, >90% of husks for *O. furnacalis*	[80]
	L1, L2, L3	*pZmUbi-1*; *Agrobacterium*-mediated	9801	-	LDR was below 2.0, >80% of kernels for *O. furnacalis*	LDR was below 1.15, >90% of husks for *O. furnacalis*	[80]
Cry1Ah	HGK60	*Ubiquitin*; *Agrobacterium*-mediated	Z 31	2.88, and 3.50 μg/g in leaves FW at 6-leaf stage, and heading stage; 3.62, and 9.98 μg/g in tassels FW at heading stage and filling stage	100% of leaves for *O. furnacalis*, >80% for *H. armigera* in 3-day bioassay	LDR was 1.29, and 2.47 for *O. furnacalis*, and *M. separata*, high resistant of kernel to *H. armigera*	[81]
	Q11, X8	*Ubiquitin*; *Agrobacterium*-mediated	Q31 × Z3	Up to 0.05% in leaves	-	LDR was 2.4 (Q11), and 3.4 (X8) for *O. furnacalis*	[82]
	G186	*Ubiquitin*; *Agrobacterium*-mediated	Z31	Up to 1 μg/g in leaves FW	100% of leaves for *O. furnacalis*	LDR was 1.3 for *O. furnacalis*	[83]
Cry1C	ZmKc-2-3	*Ubiquitin*; Gene gun-mediated	HiII	3.43, 2.71, 0.99, 0.79, 0.65, 0.66, 0.19, and 0.09 μg/g in leaves, tassel handles, stems, filaments, tassels, female ear tips, pollen, and grains FW, respectively	-	100% for *O. furnacalis*	[9]
Cry1Ie	IE09S034	*Ubiquitin*; *Agrobacterium*-mediated	Z31	-	85.42%–90.62% for *O. furnacalis*; 50% for for *H. armigera*	LDR was below 2.5 for *O. furnacalis*	[84]
Cry1Ab/2Aj	Shuangkang 12-5	*pZmUbi-1*; *Agrobacterium*-mediated	ZhengDan 958	22.80 μg/g in pollen DW	96% of whorl leaves, tassels, silks, and point of spikes, and 88% of grains for *O. furnacalis* in 7-day bioassays	100% for *O. furnacalis*	[77,85]
	N10, N20, N30, N40, N50	*Ubiquitin*; *Agrobacterium*-mediated	Hind-II	14.31–22.67 μg/g in whorl leaves DW; 20.93–49.33 μg/g in silks DW	93.2%–100% of whorl leaves, tassels, husks, silks and kernels for *O. furnacalis*	LDR was 1.0–1.50 for *O. furnacalis*	[86]
	N30	*Ubiquitin*; *Agrobacterium*-mediated	Hind-II	7.69, 5.12, 8.52, and 3.87 μg/g in whorl leaves, tassels, kernels and silks FW, respectively	100% of whorl leaves, tassels, husks, silks and kernels for *O. furnacalis*	LDR was 1.0, 100% for *O. furnacalis*	[87]
Cry1Ab/vip3DA	V3	*Ubiquitin*; *Agrobacterium*-mediated	Hind-II	4.51–9.72 μg/g in whorl leaves, tassels, husks, silks and kernels FW	100% of whorl leaves, tassels, husks, silks, and kernels for *O. furnacalis*	LDR was 1.0, 100% for *O. furnacalis*	[87]
Cry1Ah/Cry1Ie	HIF21	*Ubiquitin*; Gene gun-mediated	X090	Cry1Ah: 0.14% in leaves	-	LDR was 2.08 for *O. furnacalis*	[88]

^a^ % soluble protein (*w*/*w*) or μg/g tissues fresh weight (FW) or dry weight (DW); “-” denotes “unclear”; ^b^ Leaf damage ratings (LDR) followed the criteria described by He et al. [89], in which 1.0 = rare or sporadic pin-holes on a few leaves; 2.0 = intermediate pin-holes on a few leaves; 3.0 = many pin-holes on several leaves; 4.0 = rare or sporadic match-head-sized holes on a few leaves; 5.0 = intermediate match-head-sized holes on a few leaves; 6.0 = many match-head-sized holes on several leaves; 7.0 = rare or sporadic holes larger than match-head-sized holes on a few leaves; 8.0 = intermediate holes larger than a match-head on a few leaves, and 9.0 = many holes larger than a match-head on several leaves. The resistance-level classifications were as follow: 1.0–2.09 (highly resistant); 2.1–4.09 (resistant); 4.1–6.09 (moderately resistant); 6.1–8.09 (susceptible); and 8.1–9.0 (highly susceptible).
